# IL-6 from cerebrospinal fluid causes widespread pain via STAT3-mediated astrocytosis in chronic constriction injury of the infraorbital nerve

**DOI:** 10.1186/s12974-024-03049-z

**Published:** 2024-02-28

**Authors:** Ning Yu, Huan Cui, Sixuan Jin, Penghao Liu, Yehong Fang, Fengrun Sun, Yan Cao, Bo Yuan, Yikuan Xie, Wanru Duan, Chao Ma

**Affiliations:** 1 State Key Laboratory of Common Mechanism Research for Major Diseases, Department of Human Anatomy, Histology and Embryology, Neuroscience Center, Joint Laboratory of Anesthesia and Pain, Institute of Basic Medical Sciences Chinese Academy of Medical Sciences, School of Basic Medicine Peking Union Medical College, No. 5 DongDanSanTiao, Dongcheng District, Beijing, 100005 China; 2 Department of Neurosurgery, Xuanwu Hospital, Capital Medical University, 45# Changchun Street, Xicheng District, Beijing, 100053 China; 3 Lab of Spinal Cord Injury and Functional Reconstruction, China International Neuroscience Institute (CHINA-INI), Beijing, China; 4 Department of Psychiatry, Sir Run Run Shaw Hospital, Zhejiang University School of Medicine, Hangzhou, China; 5 National Human Brain Bank for Development and Function, Beijing, China; 6 Chinese Institute for Brain Research, Beijing, 102206 China

**Keywords:** Widespread pain, Cerebrospinal fluid, Interleukin-6, Astrocyte, Signal transducer and activator of transcription 3

## Abstract

**Background:**

The spinal inflammatory signal often spreads to distant segments, accompanied by widespread pain symptom under neuropathological conditions. Multiple cytokines are released into the cerebrospinal fluid (CSF), potentially inducing the activation of an inflammatory cascade at remote segments through CSF flow. However, the detailed alteration of CSF in neuropathic pain and its specific role in widespread pain remain obscure.

**Methods:**

A chronic constriction injury of the infraorbital nerve (CCI-ION) model was constructed, and pain-related behavior was observed on the 7th, 14th, 21st, and 28th days post surgery, in both vibrissa pads and hind paws. CSF from CCI-ION rats was transplanted to naïve rats through intracisternal injection, and thermal and mechanical allodynia were measured in hind paws. The alteration of inflammatory cytokines in CCI-ION’s CSF was detected using an antibody array and bioinformatic analysis. Pharmacological intervention targeting the changed cytokine in the CSF and downstream signaling was performed to evaluate its role in widespread pain.

**Results:**

CCI-ION induced local pain in vibrissa pads together with widespread pain in hind paws. CCI-ION’s CSF transplantation, compared with sham CSF, contributed to vibrissa pad pain and hind paw pain in recipient rats. Among the measured cytokines, interleukin-6 (IL-6) and leptin were increased in CCI-ION’s CSF, while interleukin-13 (IL-13) was significantly reduced. Furthermore, the concentration of CSF IL-6 was correlated with nerve injury extent, which gated the occurrence of widespread pain. Both astrocytes and microglia were increased in remote segments of the CCI-ION model, while the inhibition of astrocytes in remote segments, but not microglia, significantly alleviated widespread pain. Mechanically, astroglial signal transducer and activator of transcription 3 (STAT3) in remote segments were activated by CSF IL-6, the inhibition of which significantly mitigated widespread pain in CCI-ION.

**Conclusion:**

IL-6 was induced in the CSF of the CCI-ION model, triggering widespread pain via activating astrocyte STAT3 signal in remote segments. Therapies targeting IL-6/STAT3 signaling might serve as a promising strategy for the widespread pain symptom under neuropathological conditions.

**Graphical abstract:**

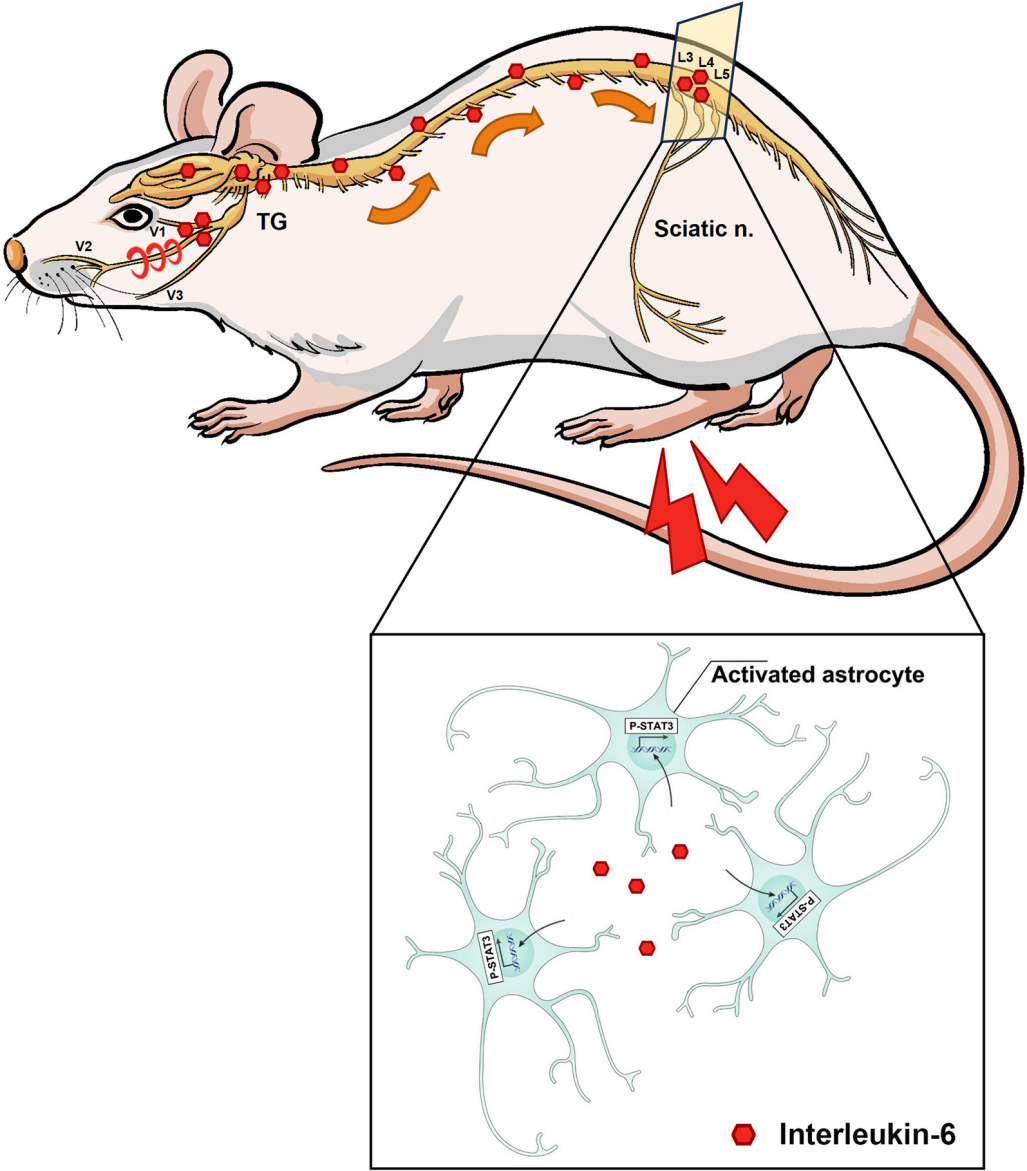

**Supplementary Information:**

The online version contains supplementary material available at 10.1186/s12974-024-03049-z.

## Background

Clinical evidence indicates that neuropathic pain manifests the feature of dissemination, often spreading to adjacent segments or symmetrical areas (mirror-image pain), and even to relatively distant body regions (widespread pain) [1, 2]. Notably, this persistent widespread pain consistently appears in a low-intensity manner but can be even more enduring than the original local pain, thereby causing long-lasting affective distress.

Preclinical studies have suggested that remote inflammatory activation evoked by the original-site neuropathy is responsible for widespread pain [3]. It has been reported that partial infraorbital nerve transection (p-IONX) induces the production of high mobility group box 1 protein (HMGB1) in the medullary dorsal horn (MDH) and lumbar spinal dorsal horn (SDH), along with activation of astrocytes and microglia. Moreover, Toll-like receptor 4 (TLR4) participates in the spread of the allodynia component of orofacial pain to distant body sites in the p-IONX model [4, 5]. However, the detailed action pattern of inflammatory signal diffusion and interaction between different spinal segments in widespread pain remains largely unexplored.

Cerebrospinal fluid (CSF), a colorless and transparent fluid existing in the ventricle and subarachnoid space, serves as lymph in the central nervous system (CNS), transporting nutrients and metabolites cyclically. The components in the CSF change dynamically under neuropathological conditions, including the infiltration of inflammatory cells and alterations of cytokines [6–9]. These factors might travel along with CSF and impact distant nervous regions, serving as messengers transmitting inflammatory signals to remote segments in widespread pain. To uncover the role of CSF in widespread pain, we here explored the cytokine messenger in the CSF along with its operating mode for remote segment neuroinflammation and widespread pain.

## Methods

### Animals

Adult male Sprague–Dawley rats (specific pathogen free, 180 g–220 g, purchased from the National Institutes for Food and Drug Control, Beijing, China) were randomly assigned to subgroups. All rats were housed at 23 °C ± 2 °C and a 12/12 h light/dark cycle-controlled room with free access to rodent chow and water. This study was approved by the Institutional Animal Care and Use Committees of the Chinese Academy of Medical Sciences and the Institute of Basic Medical Sciences (Approval Number: #211-2014).

### CCI-ION model

The chronic constriction injury of the infraorbital nerve (CCI-ION) model was established in accordance with methodologies outlined in previous studies [10]. Briefly, to induce chronic constriction injury, rats were subjected to anesthesia using pentobarbital sodium (50 mg/kg i.p.). Under anesthesia, a skin incision was made to expose the infraorbital nerve (ION) on the right side. Subsequently, four snug ligatures of chromic gut suture were loosely tied around the ION at intervals of ~ 1 mm. For graded injury severity, a portion of the ION on the right side was isolated, and 25%, 50%, or 75% of its nerve fibers were separately ligatured. In the sham operation, rats underwent only the nerve exposure without the application of ligatures.

### Behavioral test

Behavioral tests were performed in a quiet room during 7:00 am–9:00 am. Prior to testing, the rats were acclimatized for three consecutive days. The hind paw withdrawal threshold to mechanical stimuli was measured using an electronic von Frey apparatus (IITC Life Science). This electronic von Frey probe consisted of a hand-held force transducer with a fixed tungsten wire tip (200 μm diameter), applied perpendicularly to the plantar surface of the right hind paw. Thermal hyperalgesia was assessed by measuring right hind paw withdrawal thermal latency with a radiant thermal stimulator (BME-410C Plantar Test Apparatus, 50W), as previously described [11, 12]. We set a 20 s cutoff time for each thermal stimulus and a 5 min interval time between two adjacent stimuli, in order to avoid potential damage. For hind paw mechanical threshold and thermal latency, three repeated measurements were performed for each subject with a 5 min interval, and the threshold was defined as the mean of the three readings.

The mechanical sensitivity of the vibrissa pad, the infraorbital nerve-innervating region, was assessed using a set of calibrated von Frey filaments ranging from a minimal force of 0.008 g to a maximum of 15 g. To observe the behavioral response stimulated by filaments, each rat was placed in a wire cage (25 × 10 × 10 cm), and von Frey mechanical stimuli were applied to the orofacial skin near the center of the right vibrissa pad at least 3 times. As reported in previous studies, behavioral indications of nociception included: (1) immediate head withdrawal and subsequent repeated face wiping directed to the stimulated facial area; (2) aggressive actions such as attacking, biting or grabbing the filament; and (3) avoidance movements, including escaping from the filament to avoid further mechanical stimulation [13]. The lowest filament force eliciting a definitive nociceptive reaction was defined as the vibrissa pad mechanical threshold.

The pain-related behavior in the CCI-ION model was observed on the 0th, 7th, 14th, 21st, and 28th days post surgery. For the CSF transplanted model, behavior assessments were conducted on the 0th, 1st, 3rd, 5th, 7th, 8th, 9th, 10th days following the procedure. Each reagent applied was previously prepared and then coded by a technician, which was blinded to the experimenters. For each behavior assay, the experimenters who performed animal model and reagents injection were separated with behavioral testers, therefore behavioral testers were blinded to the animal surgery treatment (sham or CCI-ION) and reagents injection.

### Antibody array

The Rat cytokine array (AAR-CYT-G2-8; Ray Biotech, Norcross, GA, USA) was employed following the manufacturer’s guidelines to quantify the levels of 34 cytokines in CSF samples collected on postoperative day (POD) 14. Four samples each from the sham group (labeled Sham 01 to Sham 04) and CCI-ION group (labeled CCI-ION 01 to CCI-ION 04) were analyzed. Positive signals were detected on glass chips using a GenePix 4000B Microarray Scanner (Molecular Devices, Sunnyvale, CA, USA). Fluorescence intensities were normalized against internal positive controls. Cytokines were screened under integrated conditions: the CCI-ION group compared to the sham group (p < 0.05) for samples with fluorescence intensity values. Differentially expressed proteins were organized using hierarchical clustering and visualized in a heat map format. Enrichment analysis was performed using R software (http://www.r-project.org/).

### Reverse transcription and quantitative RT-PCR

MDH and ipsilateral trigeminal ganglia (TG) were harvested on POD 14 from both sham and CCI-ION rats and flash-frozen in liquid nitrogen. Total RNAs were extracted using Trizol reagent (Invitrogen, Grand Island, NY, USA) and reverse transcribed using PrimeScript™ RT Master Mix (Takara, Japan), following the manufacturer’s protocol. Quantitative RT-PCR (qRT-PCR) analyses were conducted on a Bio-Rad CFX96 machine using SYBR Premix Ex Taq (Takara, Japan) (Primers: IL-6, Forward, TGATGGATGCTTCCAAACTG; Reverse, GAGCATTGGAAGTTGGGGTA and β-Actin, Forward, CACCCGCGAGTACAACCTTC; Reverse, CCCATACCCACCATCACACC). The expression levels of target genes were quantified relative to the level of β-Actin gene expression using the 2^−ΔΔCT^ method. Real-time PCR experiments for each gene were replicated three times.

### ELISA

The total IL-6 levels in rats’ CSF were assessed using the rat IL-6 ELISA kit (Elabscience, E-EL-R0015). Briefly, a Corning Costar 9018 ELISA plate was coated with capture antibody and incubated overnight at 4 °C. After blocking the coated wells with Blocking Buffer for 1 h at room temperature, CSF samples were added following a 100-fold dilution. Detection of total IL-6 was achieved using an HRP-conjugated anti-rat IL-6 monoclonal antibody. Then, Streptavidin-HRP monoclonal antibody was applied as a second-step reagent.

### Intracisternal injection and CSF collection

The intracisternal (i.c.) transplantation of CSF and drug administration into the medulla segment were conducted as previously described [14, 15]. In brief, we utilized a custom-made syringe with a 45° angled, length-limited (4.0 mm) needle. During CSF transplantation, 15 μl of CSF from either sham or CCI-ION model rats was injected on each occasion, with a total of four injections. To neutralize IL-6 in the CCI-ION CSF prior to injection, rabbit anti-IL-6 antibody (abcam, ab6672, 0.05 μg/μL) was mixed with the CSF and incubated at 37 °C for 1 h. As a control, rabbit isotype IgG was used. The applied drugs included rabbit isotype IgG (CST, 3900, 0.25 μg/μL), anti-IL-6 antibody (abcam, ab6672, 0.1 μg/μL and 0.25 μg/μL), and IL-6 protein (1.0 μg and 5.0 μg), with each injection volume adjusted to 5.0 μL.

For CSF collection, rats were anesthetized with pentobarbital sodium (50 mg/kg i.p.). A skin incision was made above the epencephalon to expose the foramen magnum. CSF were carefully drawn out using an insulin needle, appearing as a yellowish transparent liquid. Collected CSF samples were immediately flash-frozen with dry ice and subsequently stored at − 80 °C until required for analysis or application.

### Intraspinal injection

The procedure for intraspinal (i.s.) injection in rats was adapted from established protocols [16]. Briefly, rats underwent deep anesthesia using isoflurane. A laminectomy was meticulously performed at the lumbar level of the spinal cord. The spinal cord was then stabilized in a stereotactic frame using specialized spinal clamps. For the injection process, a fine glass micropipette was carefully positioned 0.5 mm lateral to the midline of the spinal cord, penetrating to a depth of 0.5 mm. The injection speed was controlled at a rate of 80 nL/min. Targeting the L3-L5 spinal segments, we systematically injected 200 nl of the drug solution into four precisely chosen sites. The drug concentration used were as follows: Minocycline (Sigma, M9511) at 1.0 μg/μL in PBS, LAA (Sigma, A7275) at 10.0 nmol/μL in PBS, STAT3-IN-3 (MedChemExpress, HY-128588) at 50.0 μM in 1% DMSO. The micropipette was maintained in position for an additional 5 min before slowly withdrawal to ensure optimal delivery.

### Primary astrocyte culture

Primary astrocytes were dissected from the brain cortices of neonatal rats. The meninges were carefully removed, and cortical tissues were gently triturated with a pipette. The cell suspension was centrifuged at 1200 rpm for 3 min and subsequently resuspended in Dulbecco’s modified eagle medium (DMEM, Gibco), supplemented with 10% fetal bovine serum (FBS, Gibco) and 1% penicillin/streptomycin (Gibco). Cultures were then incubated at 37 °C in a humidified atmosphere containing 95% air and 5% CO_2_. Upon reaching 95% confluence, differentiation was induced using dibutyryl cAMP (0.15 mM, Sigma–Aldrich). To purify the astrocyte monolayer, contaminating cells were removed by shaking the culture overnight at 220 rpm and 37 °C. To stimulate the cultured astrocytes with CSF, collected CSF was integrated into complete medium at a 2% concentration (2% CSF + 98% complete medium), and astrocytes were incubated with this mixture for 12 h prior to sample collection. To neutralize IL-6 in CCI-ION CSF, CSF samples were pre-treated with rabbit anti-IL-6 antibody (abcam, ab6672, 0.05 μg/μL) at 37 °C for 1 h, with rabbit isotype IgG (CST, 3900, 0.05 μg/μL) serving as a control.

### Calcium imaging

For calcium imaging, primary astrocytes were loaded with Fura 2-acetoxy-methyl ester (10 μM, invitrogen) in darkness for 45 min at 37 °C. The cells were then transferred to a recording chamber (volume: 1.0 mL), continuously perfused with HEPES buffer at a flow rate of 1.5 ml/min at room temperature. CSF from either CCI-ION rats or sham rats was applied via bath application (100 μL). To ascertain astrocyte viability, HEPES containing 50 mM K^+^ was introduced at the end of each experiment. Fluorescence signals at 340 and 380 nm excitations were recorded every 2 s using an upright NIKON ECLIPSE Ti microscope equipped with a ratiometric imaging system (Nikon NIS-Elements AR 4.00.00, Japan). The ratio of fluorescence intensity at 340 nm and 380 nm [F_(340/380)_] within a certain astrocyte was used to infer relative intracellular calcium concentration.

### Immunohistochemistry

#### Immunohistochemistry for cryosections

Immunofluorescence was performed as Su et al. described [17]. Rats were anesthetized using an intraperitoneal injection of sodium pentobarbital (50 mg/kg) and subsequently underwent transcardial perfusion with sterile phosphate-buffered saline (PBS), followed by ice-cold 4% paraformaldehyde (Sigma, USA). Spinal cords segments C3–C5, T6–T8, and L4–L6 were extracted and post-fixed in 4% paraformaldehyde for 4 h at 4 °C before dehydration in 30% sucrose. The tissues were then embedded in OCT compound (Tissue-Tek, Japan) and serially sectioned into 12 μm-thickness slices using a cryostat (Leica 2000, Germany). Sections were permeabilized in 0.3% Triton X-100 for 1 h at 37 °C and blocked with 10% donkey serum for 1 h. They were then incubated overnight with primary antibodies at 4 °C in a humidified chamber. After washing with PBS, sections were treated with corresponding secondary antibodies for 1 h at room temperature, followed by another PBS wash and coverslipping using a DAPI-containing Mounting Medium (ZSJB-Bio, Beijing, China). Images were captured using a laser confocal microscopy system (FV1000 and Olympus FluoView software, Olympus, Japan).

#### Immunohistochemistry for primary astrocyte cultures

Following pharmacological treatment, astrocytes cultured on glass coverslips were fixed in cold 4% paraformaldehyde (Servicebio, Wuhan, China) for 10 min. The cells were then permeabilized using 0.2% Triton X-100 for 15 min and blocked with 3% bovine serum albumin (BSA) for 20 min. Overnight incubation with primary antibodies was performed at 4 °C. Following this, the coverslips were washed with PBS and incubated with the appropriate secondary antibodies for 1 h. The final step involved mounting the coverslips using a DAPI-containing Mounting Medium (ZSJB-Bio, Beijing, China). Images were acquired and analyzed with a Leica TCS-SP8 STED 3X laser confocal microscope (Leica, Germany). Additional file 2: Table S1 lists the primary and secondary antibodies used for immunofluorescence staining analysis.

### Statistical analysis

All data were presented as group mean and its standard error (mean ± SEM). Shapiro–Wilk test was applied to test the normality of each data set for parametric test. Student’s t test was used to detect differences between two groups, while one-way analysis of variance (ANOVA) followed by the Bonferroni post hoc test was used to detect the difference between multiple groups. For the behavioral test, two-way repeated measures ANOVA was used: one factor was the time point, and the other was the treatment of the rats. All statistical analyses were performed in GraphPad Prism for Windows version 7.0. A statistically significant difference was defined as a two-sided p value < 0.05. The t value and F value from our statistical tests were detailed in Additional file 3: Table S2.

## Results

### Transplantation of CCI-ION’s CSF induced widespread pain in naïve rats

**Fig. 1:**
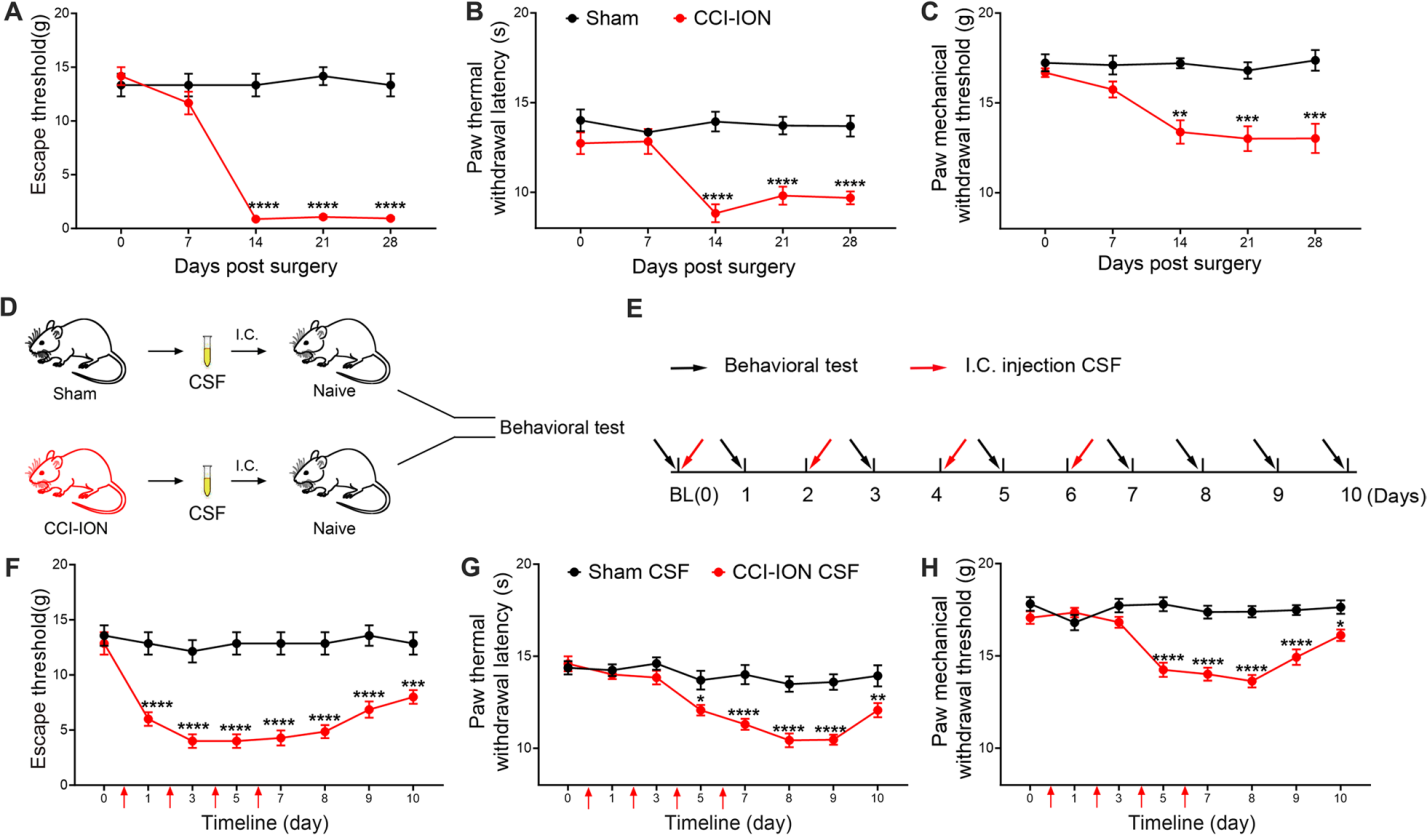
Widespread pain was induced by CCI-ION rats’ CSF. **A** The escape threshold of vibrissal pad to mechanical stimuli in the sham and CCI-ION groups. Two-way ANOVA, ****p < *0.0001*, n = 6/group. **B** The paw withdrawal threshold to thermal stimuli in the sham and CCI-ION groups. Two-way ANOVA, ****p < *0.0001*, n = 6/group. **C** The paw withdrawal threshold to mechanical stimuli in the sham and CCI-ION groups. Two-way ANOVA, **p < *0.01*, ***p < *0.001*, n = 6/group. **D** The schematic diagram of CSF transplantation. **E** The workflow of i.c. injection CCI-ION rats’ or sham rats’ CSF and behavioral tests. **F** The mechanical escape threshold of vibrissal pad in rats receiving sham CSF or CCI-ION CSF. Two-way ANOVA, ***p < *0.001*, ****p < *0.0001*, n = 7/group. **G** The thermal withdrawal threshold of hind paw in rats receiving sham CSF or CCI-ION CSF. Two-way ANOVA, *p < *0.05*; **p < *0.01*; ****p < *0.0001*, n = 7/group. **H** The mechanical allodynia of hind paw in rats receiving sham CSF or CCI-ION CSF. Two-way ANOVA, *p < *0.05*; ****p < *0.0001*, n = 7/group

Compared with the sham group, the mechanical escape threshold in the vibrissal pad of CCI-ION rats was significantly decreased from POD 14 to POD 28, suggesting that CCI-ION induced neuropathic pain in the region innervated by the injured infraorbital nerve (Fig. 1A). Strikingly, the thermal withdrawal latency of the hind paw was significantly decreased in CCI-ION rats from POD 14 to POD 28, as well as the hind paw mechanical withdrawal threshold (Fig. 1B, C), indicating the induction of widespread pain in uninjured segments of the CCI-ION model. To further investigate the role of CSF in widespread pain, we collected the CSF from CCI-ION or sham rats on POD 14 and transplanted it to naïve rats through i.c. injection (Fig. 1D, E). The escape threshold of the vibrissal pad in rats receiving sham’s CSF showed no significant difference as compared with the baseline. However, the rats receiving CCI-ION’s CSF, compared with those receiving sham’s CSF, presented mechanical allodynia in the vibrissal pad which could last to the 4th day after the last CSF injection (Fig. 1F). In addition, the thermal hyperalgesia and mechanical allodynia in the hind paw developed after three times CSF transplantation and maintained to 4th day after the last CSF transplantation in rats adopting CCI-ION’s CSF, which could not be observed in rats treated by sham’s CSF (Fig. 1G, H). These above data indicated that the “neuropathic” CSF might serve as the source of widespread pain, transmitting nociceptive signals to corresponding body regions of uninjured spinal segments.

### IL-6 from CSF mediated widespread pain in CCI-ION model

**Fig. 2:**
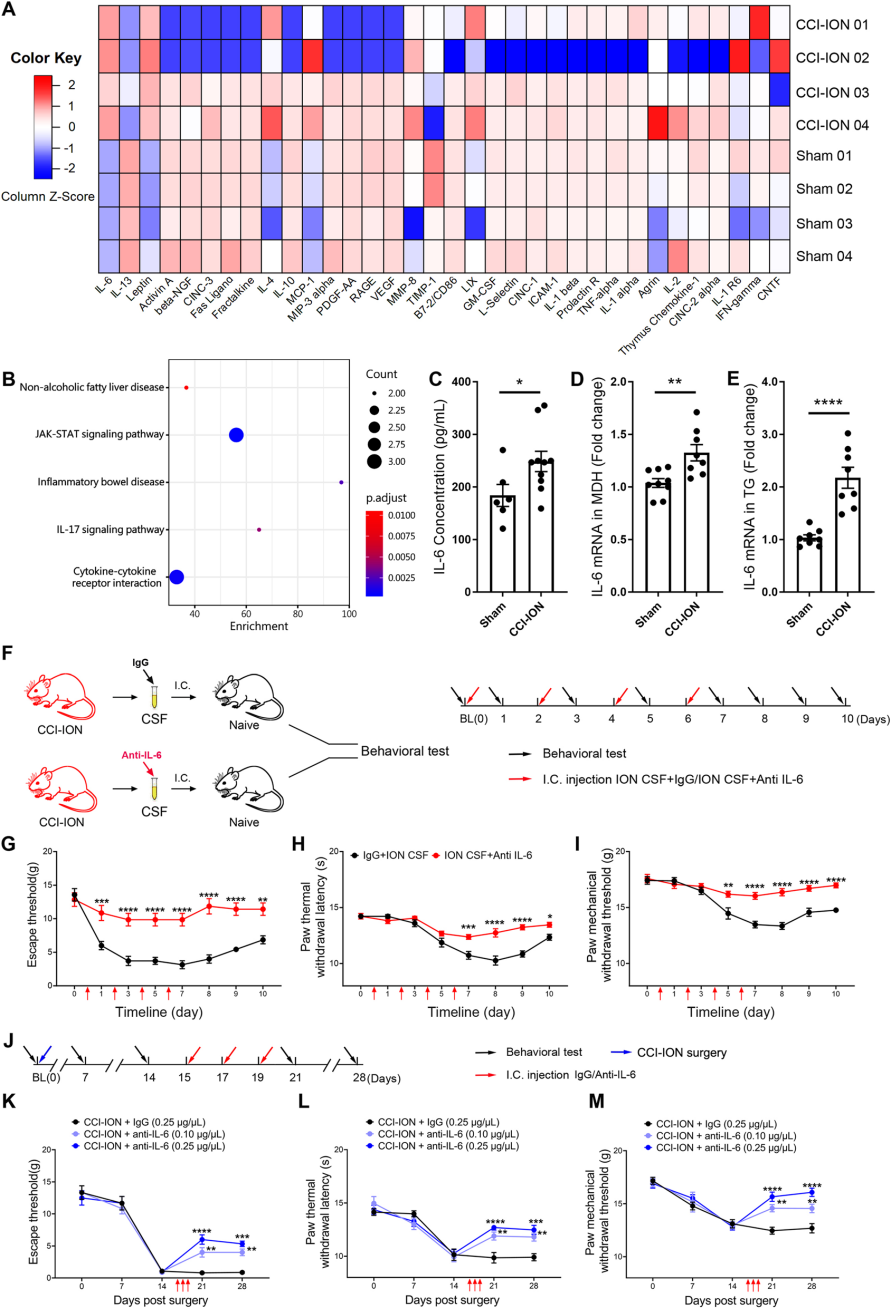
IL-6 from CSF mediated widespread pain. **A** Heatmap showed the alteration of all tested 34 cytokines in CCI-ION’s CSF compared with the sham’s CSF. n = 4/group. **B** The KEGG analysis for the altered cytokines in the CSF of CCI-ION group. **C** The ELISA analyzed the concentration of IL-6 in the CSF samples from CCI-ION group and sham group. t test, *p < *0.05*, n = 6–10/group. **D** qRT-PCR revealed a significant increase in mRNA expression level of IL-6 in MDH after CCI-ION. t test, **p < *0.01*, n = 8–9/group. **E** qRT-PCR revealed a significant increase in mRNA expression level of IL-6 in TG after CCI-ION. t test, ****p < *0.0001*, n = 8/group. **F** The workflow for i.c. injection of CCI-ION CSF pre-treated by IL-6 antibody or isotype IgG and relative behavioral test for recipient rats. **G** The time course of mechanical escape threshold of vibrissal pad for recipient naïve rats receiving CCI-ION CSF pre-incubated by IL-6 antibody or isotype IgG. Two-way ANOVA, **p < *0.01*, ***p < *0.001*, ****p < *0.0001*, n = 7/group. **H** The thermal withdrawal latency of hind paw for recipient naïve rats receiving CCI-ION CSF pre-incubated by IL-6 antibody or isotype IgG. Two-way ANOVA, **p < *0.01*; ****p < *0.0001*, n = 7/group. **I** The mechanical threshold of hind paw for recipient naïve rats receiving CCI-ION CSF pre-incubated by IL-6 antibody or isotype IgG. Two-way ANOVA, **p < *0.01*, ****p < *0.0001*, n = 7/group. **J** The workflow for application of different concentrations of IL-6 antibody or isotype IgG for CCI-ION rats. **K** The escape threshold of vibrissal pad in CCI-ION rats receiving different concentrations of IL-6 antibody or isotype IgG. Two-way ANOVA, **p < *0.01*, ***p < *0.001*, ****p < *0.0001*, vs. IgG, n = 6/group. **L, M** The thermal withdrawal latency and mechanical threshold of hind paw in CCI-ION rats receiving IL-6 antibody or isotype IgG. Two-way ANOVA, **p < *0.01*, ***p < *0.001*, ****p < *0.0001*, vs. IgG, n = 6/group

To further determine the role of CSF in widespread pain, we utilized an antibody array to scan the alteration of CSF in the CCI-ION model. Among the 34 tested factors and cytokines, interleukin-6 (IL-6) and leptin were elevated in CCI-ION’s CSF on POD 14, while interleukin-13 (IL-13) was significantly decreased in CCI-ION’s CSF compared with sham’s CSF (Fig. 2A). Furthermore, KEGG analysis revealed that pathways, especially the Janus-activated kinase/signal transducer activator of transcription (JAK/STAT) signaling pathway and cytokine-cytokine receptor interaction, were significantly enriched within the altered factors (Fig. 2B). To further validate the antibody array data, IL-6 ELISA was applied, showing a significant increase in IL-6 in the CSF from CCI-ION rats (Fig. 2C). Additionally, CCI-ION surgery upregulated IL-6 mRNA expression in the medullary dorsal horn (MDH) and trigeminal ganglion (TG), serving as the potential source of CSF IL-6 (Fig. 2D, E). Subsequently, i.c. injection of IL-6 protein in naïve rats verified its nociceptive effect, inducing orofacial mechanical allodynia together with mechanical allodynia and thermal hyperalgesia in hind paw (Additional file 1: Fig. S1A–D). To ascertain if CSF IL-6 was the main element triggering widespread pain, IL-6 antibody was applied to neutralize IL-6 in the CSF of CCI-ION model. The CCI-ION CSF, collected from CCI-ION rats on POD 14, was pre-incubated with IL-6 antibody or isotype IgG before being injected into the cisterna of recipient rats (Fig. 2F). The following behavioral assay showed that neutralizing CSF IL-6 significantly alleviated pain-like behavior in both vibrissal pad and hind paw induced by CCI-ION CSF (Fig. 2G, I). Furtherly, we investigated if IL-6 antibody injection into CCI-ION model could alleviate the original neuropathic pain in vibrissal pad and widespread pain in hind paw. The behavioral assay showed that i.c. injection of IL-6 antibody mitigated both mechanical allodynia in the vibrissal pad and widespread pain in the hind paw in a dosage-dependent manner (Fig. 2J–M). Collectively, CSF IL-6 served as a key mediator triggering widespread pain in remote spinal segments.

### Intensity theory model of nerve injury extent, CSF IL-6 content, and widespread pain

**Fig. 3:**
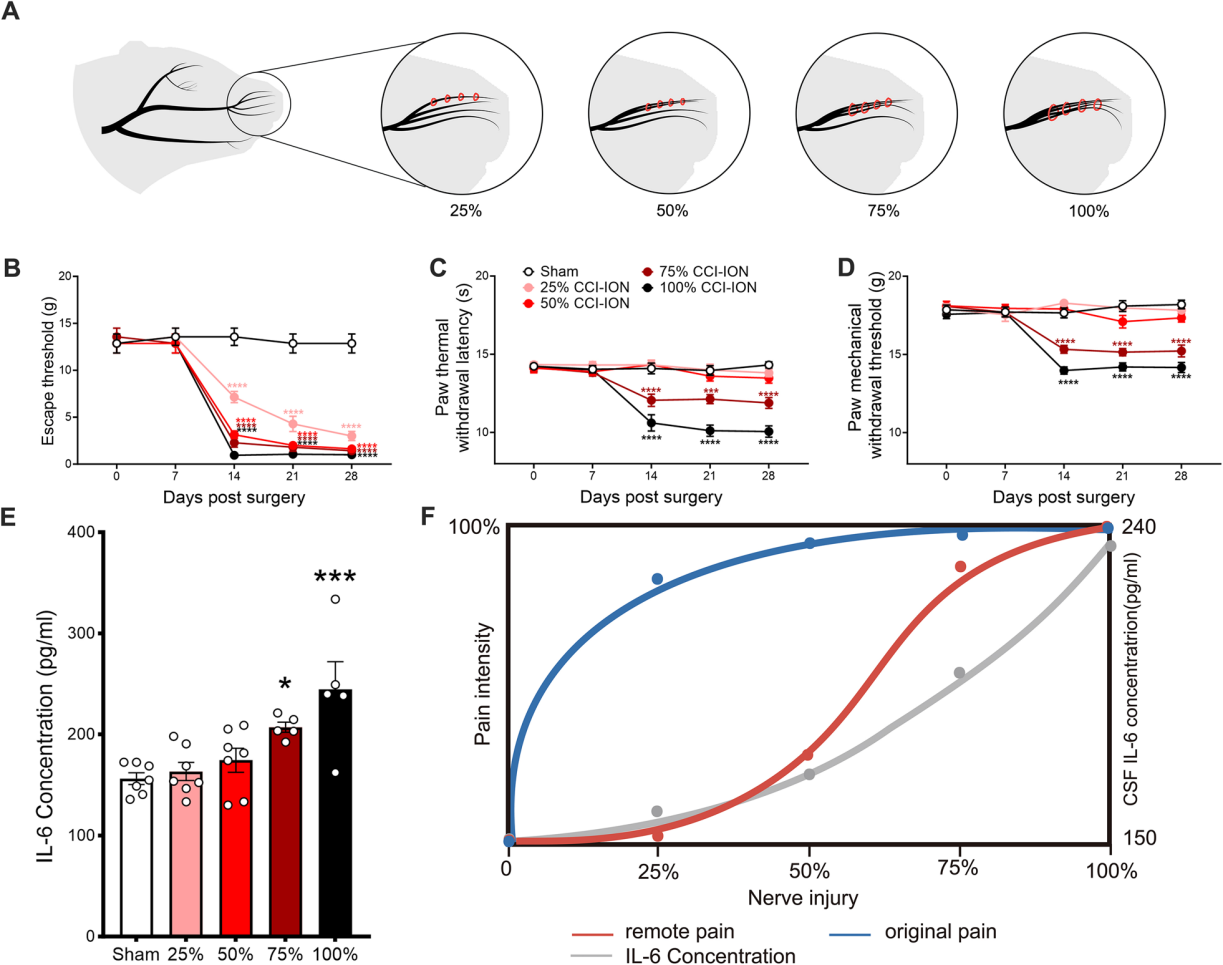
Intensity theory model of nerve injury extent, CSF IL-6 content, and widespread pain. **A** The schematic diagram of 25%, 50%, 75%, and 100% ligation of infraorbital nerve. **B** The time course of mechanical escape threshold of vibrissal pad of CCI-ION rats with different degrees of ligation. Two-way ANOVA, ****p < *0.0001*, vs. sham, n = 7/group. **C-D** The paw thermal withdrawal latency and mechanical threshold in CCI-ION rats with different degrees of infraorbital nerve ligation. Two-way ANOVA, ***p < *0.001*, ****p < *0.0001*, vs. sham, n = 7/group. **E** ELISA analyzed the concentration of IL-6 in the CSF samples from the sham rats and CCI-ION rats with different degrees of nerve ligation. One-way ANOVA, *p < *0.05*, ***p < *0.001*, vs. sham, n = 5–7/group. **F** The sketch of an intensity theory model: the relationship of nerve injury, IL-6 concentration in the CSF and widespread pain

Given that widespread pain was not observed in any neuropathological condition, we proposed an intensity theory model indicating that CSF IL-6 concentration correlated with the extent of nerve injury and gated the occurrence of widespread pain. To validate this hypothesis, we ligatured 25%, 50%, 75%, and 100% fibers of the infraorbital nerve to simulate nerve injury from mild to heavy intensity (Fig. 3A). The behavioral test showed that infraorbital nerve injury intensity from 25 to 100% triggered mechanical allodynia in the vibrissal pad, while infraorbital nerve injury could not induce widespread pain in the hind paw at intensities lower than 75%. Moreover, 75% infraorbital nerve injury significantly contributed to thermal hyperalgesia and mechanical allodynia in the hind paw, which intensified as the injury intensity increased to 100% (Fig. 3B–D). To examine the relationship between nerve injury intensity and CSF IL-6, we collected CSF from rats receiving gradient infraorbital nerve injury on POD 14 and performed an ELISA test for IL-6. The results showed that nerve injury from 75 to 100% significantly increased the IL-6 content in the CSF compared with the sham group, consistent with the occurrence of widespread pain (Fig. 3E). The sketch illustrated that IL-6 concentration in the CSF gated remote pain occurrence, gradually increasing with nerve injury amplification (Fig. 3F).

### Recipient astrocytes in remote segments mediated widespread pain in CCI-ION model

**Fig. 4:**
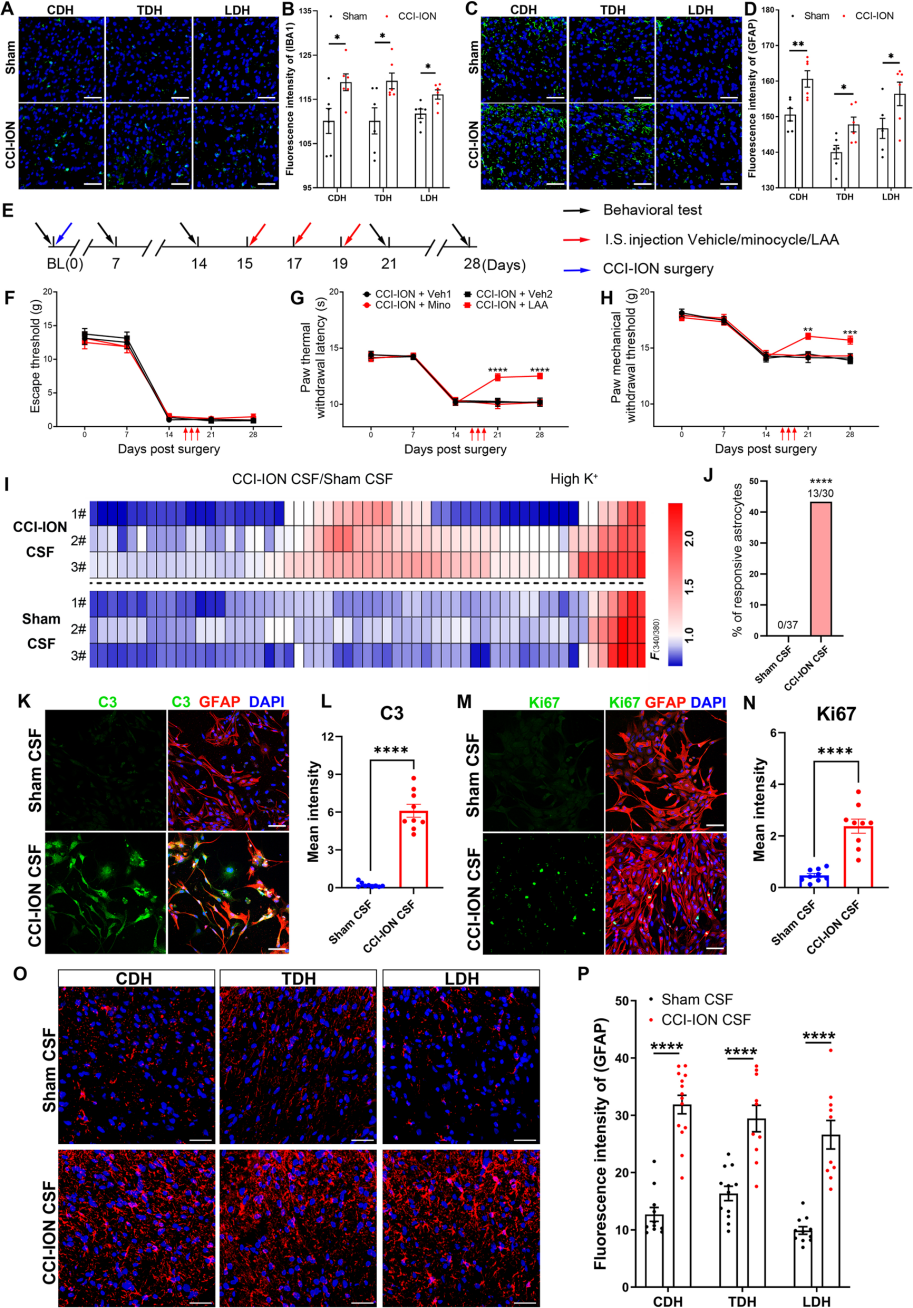
Astrogliosis in SDH of remote segments mediated widespread pain. **A, B** Immunofluorescence staining showed increased IBA1 expression in CDH, TDH, and LDH of CCI-ION rats. Scale bar: 50 μm. t test, *p < *0.05*, n = 6/group. **C, D** Immunofluorescence staining showed increased GFAP expression in CDH, TDH, and LDH of CCI-ION rats. Scale bar: 50 μm. t test, *p < *0.05*, **p < *0.01*, n = 6/group. **E** The workflow of intraspinal application LAA, minocycline or vehicle. **F** The time course of mechanical escape threshold of vibrissal pad for CCI-ION rats receiving LAA, minocycline or vehicle. Two-way ANOVA, n = 8/group. **G, H** The paw thermal withdrawal latency and mechanical withdrawal threshold in CCI-ION rats receiving LAA, minocycline or vehicle. Two-way ANOVA, **p < *0.01*, ***p < *0.001*, ****p < *0.0001*, n = 8/group. **I** Heatmap showed Ca^2+^ response of primary cultured astrocytes to CSF from CCI-ION rats and sham rats. **J** Quantitative analysis showed the percentage of responsive astrocytes after addition of CSF from CCI-ION rats and sham rats. Chi-square test, ****p < *0.0001*. **K**, **L** Immunofluorescence staining of C3 in primary cultured astrocytes after incubation of sham’s CSF and CCI-ION’s CSF. t test, ****p < *0.0001*, n = 9/group. Scale bar: 75 μm. **M**, **N** Immunofluorescence staining of Ki67 in primary cultured astrocytes after incubation of sham’s CSF and CCI-ION’s CSF. t test, ****p < *0.0001*, n = 9/group. Scale bar: 75 μm. **O**, **P** Immunofluorescence staining showed GFAP expression in CDH, TDH, and LDH of rats receiving sham’s CSF or CCI-ION’s CSF. t test, ****p < *0.0001*. n = 10–14/group. Scale bar: 50 μm

We next examined the responsive cell type to CSF IL-6 in remote segments, with astrocytes and microglia as main candidates given their neuroinflammatory characteristics. Upregulated expression of microglia marker IBA1 and astrocyte marker GFAP in cervical spinal dorsal horn (CDH), thoracic spinal dorsal horn (TDH), and lumbar spinal dorsal horn (LDH) was detected by immunofluorescence staining (Fig. 4A–D). To investigate the role of glia, we applied i.s. injection of the astrocyte inhibitor L-2-Aminoadipic acid (LAA) and microglia inhibitor minocycline (Mino) to LDH. The microinjection was applied on POD 15, and tissues were collected on POD 28 (Fig. 4E). The behavioral assay showed no significant difference among groups before microinjection. After microinjection of LAA or Mino, the mechanical escape threshold in the vibrissal pad did not change on POD 21, nor POD 28 (Fig. 4F). However, LAA injection significantly alleviated mechanical allodynia and thermal hyperalgesia of the hind paw, compared with its vehicle, while Mino failed to mitigate widespread pain (Fig. 4G, H). The above data suggested that astrocytes in SDH of remote segments mediated widespread pain. Additionally, we applied calcium imaging for cultured astrocytes stimulated by CSF from sham or CCI-ION rats to determine if CSF could directly activate astrocytes. The heat map showed that CCI-ION CSF, but not sham CSF, induced prominent calcium influx in astrocytes, presenting the direct effects of CCI-ION CSF on astrocytes in vitro (Fig. 4I, J). Moreover, primary astrocytes co-incubated with CSF from CCI-ION rats exhibited increased expression of complement 3 (C3) and Ki67, suggesting the excessive activation and proliferation of astrocytes induced by CCI-ION’s CSF (Fig. 4K–N). To further validate if widespread pain occurs due to the interaction between CSF and remote astrocytes, we applied CSF transplantation into the cisterna magna and detected GFAP expression in CDH, TDH, and LDH. The immunofluorescence results showed that CCI-ION CSF transplantation upregulated GFAP expression in remote segments (Fig. 4O, P), suggesting that CCI-ION CSF evoked astrocytosis. Taken together, CSF could directly challenge astrocytes in remote segments, mediating widespread pain.

### Astrocytic STAT3 activation in the SDH of remote segments is necessary for widespread pain

**Fig. 5:**
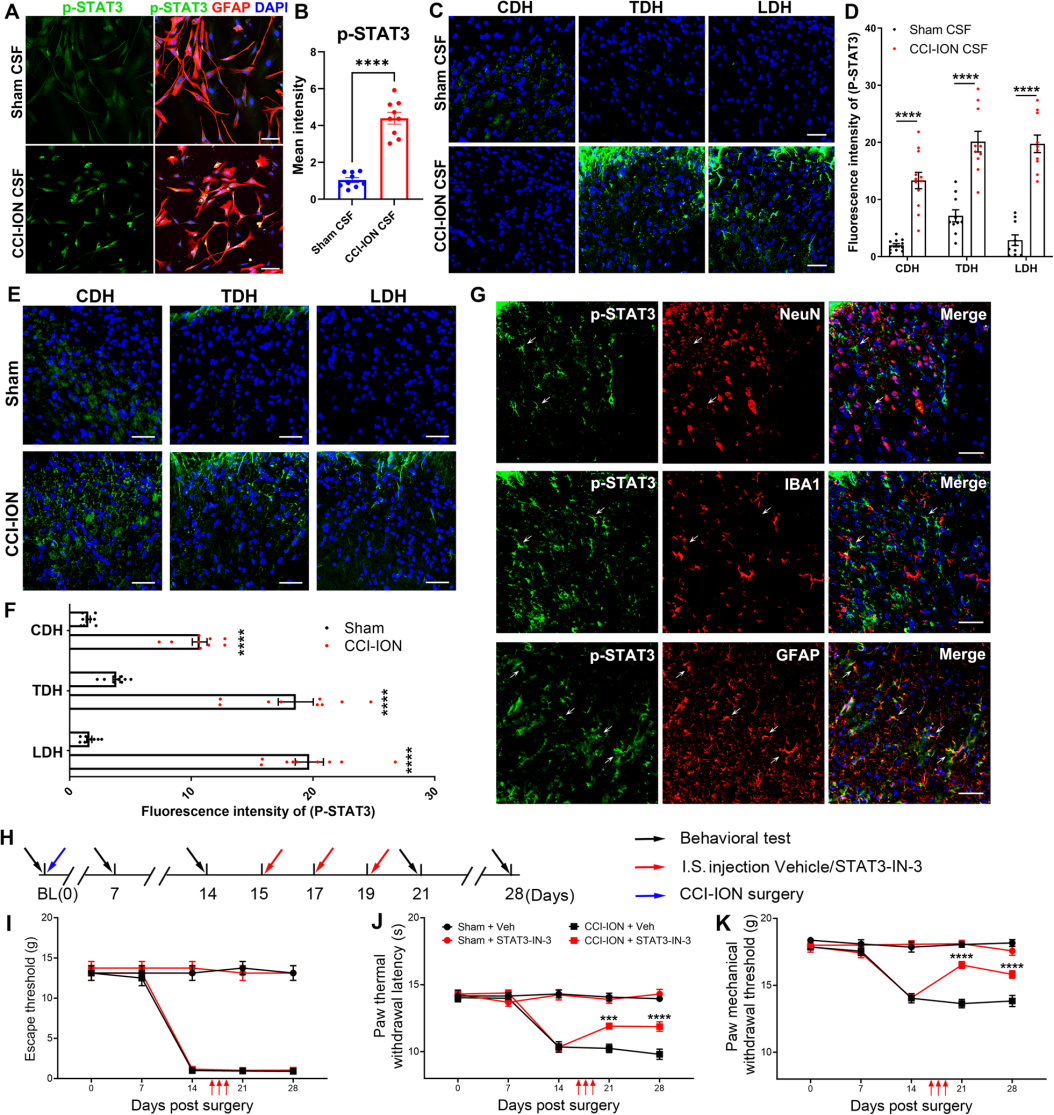
Activation of astrocytic STAT3 in SDH of remote segments was necessary for widespread pain. **A, B** Immunofluorescence staining of p-STAT3 in primary cultured astrocytes after incubation of sham’s CSF and CCI-ION’s CSF. t test, ****p < *0.0001*, n = 9/group. Scale bar: 75 μm. **C, D** Immunofluorescence staining of p-STAT3 expression in CDH, TDH, and LDH of rats receiving sham rats’ CSF or CCI-ION rats’ CSF. t test, ****p < *0.0001*, n = 10–12/group. Scale bar: 50 μm. **E, F** Immunofluorescence staining showed upregulated p-STAT3 expression in CDH, TDH, and LDH of CCI-ION rats. t test, ****p < *0.0001*, n = 9/group. **G** Representative images of double immunofluorescence staining of p-STAT3 and NeuN, IBA1 or GFAP in dorsal horn of CCI-ION rats. Scale bar: 50 μm.** H** The workflow for intraspinal injection of STAT3-IN-3 or vehicle in CCI-ION rats. **I** The time course of mechanical withdrawal threshold of vibrissal pad for CCI-ION rats receiving injections of STAT3-IN-3 or vehicle. Two-way ANOVA, n = 8/group. **J, K** The paw thermal withdrawal latency and mechanical withdrawal threshold in CCI-ION rats receiving injections of STAT3-IN-3 or vehicle. Two-way ANOVA, ***p < *0.001*, ****p < *0.0001*, n = 8/group

Previous studies have demonstrated the role of signal transducer and activator of transcription 3 (STAT3) in downstream cellular events in response to IL-6. Combined with the KEGG analysis we obtained, we further detected the expression of STAT3-phospho Y705 (p-STAT3), the activated formation of STAT3, in primary cultured astrocytes. It was observed that the astroglial expression of p-STAT3 was significantly upregulated after incubation with CCI-ION’s CSF (Fig. 5A, B). Moreover, the upregulated expression of C3, Ki67, and p-STAT3 in primary astrocytes induced by CCI-ION’s CSF were significantly suppressed by IL-6 antibody, indicating the key role of CSF IL-6 in activating astrocyte in vitro (Additional file 1: Fig. S2A–F). In addition, the p-STAT3 signaling was examined at different segmental levels of SDH in naïve rats receiving sham CSF or CCI-ION CSF. As treated by CSF from sham rats, limited signal of p-STAT3 in the SDH of naïve rats was detected. Nevertheless, i.c. injection of CCI-ION’s CSF induced the expression of p-STAT3 in CDH, TDH, and LDH (Fig. 5C, D). Similarly, enhanced fluorescence intensity of p-STAT3 was also observed in remote segments of SDH from CCI-ION rats (Fig. 5E, F). Immunofluorescence staining further distinguished that p-STAT3 co-localized with GFAP^+^ astrocyte in LDH from CCI-ION model. Moreover, neither the IBA1^+^ microglia nor the NeuN^+^ neuron co-expressed p-STAT3, suggesting that the phosphorylation and activation of astroglial STAT3 were specifically induced in the remote spinal segment astrocyte of CCI-ION model (Fig. 5G). The role of p-STAT3 in widespread pain was further determined by behavioral assay. We applied i.s. injection of STAT3-IN-3, the antagonist of p-STAT3, into LDH of CCI-ION rats and evaluated the pain-like behavior in vibrissal pad and hind paw (Fig. 5H). The mechanical allodynia in vibrissal pad remained unchanged, while the widespread pain in the hind paw was partially ameliorated (Fig. 5I–K), suggesting the potential therapeutic effect of p-STAT3 inhibitor in widespread pain.

## Discussion

Recent studies have revealed the dissemination character of neuropathic pain, referring to pain symptom occurring at distant segments beyond the corresponding segment of the original neuropathy. This systemic effect is notorious and stubborn, significantly reducing patients’ quality of life [18–21]. In this study, we first demonstrate that the CSF from CCI-ION is “painful”, diffusing mechanical and thermal hypersensitivity to naïve rats. This phenomenon suggests that CSF might act as a medium, transferring pain signals from the initial injured segment to distant non-injured spinal segments via its circulating flow within the CNS.

In fact, the composition of CSF is not fixed and changes dynamically under neuropathological conditions, such as the occurrence Aβ of and tau protein in Alzheimer’s disease (AD) and the enrichment of α-synuclein in Parkinson’s disease [22–27]. Moreover, CSF contains inflammatory cytokines under multiple neuropathological conditions, which serve as an “accomplice” during disease progression [28–30]. These novel observations in our present study highlight the long-term and systemic effects of nerve-involved tissue damages, such as surgery history and cancer invasion, on neuroinflammation in the CNS, mediated by CSF inflammatory cytokines. It has also been reported that pain, especially fibromyalgia and postherpetic neuralgia, is related to other neurological disorders including AD and emotional disorders [31–33]. These studies suggest that the diffusion of inflammatory factors transported by the CSF might seed neuroinflammation and contribute to pain-related systemic dysfunction in the CNS. This concept is consistent with the current clinical note which has listed surgery history as an independent risk factor for various neuropathological diseases including AD [34–36]. Therefore, pain serves as multiple warnings, not only for escaping dangers in the present but also for potential neurological disorders in the future [37, 38]. Moreover, our study indicates that the occurrence of widespread pain is gated by the extent of nerve injury and related to CSF IL-6. These results underline the “the less, the better” principle of nerve injury for widespread pain, even if the pain symptom might not noticeably worsen in the original injured spinal segment. In general, actively controlling the degree of nerve damage and targeting CSF IL-6 therapy are optional strategies to mitigate the systemic effects of tissue trauma and nerve damages [39]. Notably, we can not reach the conclusion that only severe injuries (75% or higher) cause CSF IL-6 elevation, even if the elevated CSF IL-6 was detected in 75% and 100% infraorbital nerve injury on POD 14. The CSF IL-6 concentration might be complicated in neuropathological process. Relatively slight nerve injury might also induce temporary IL-6 release into CSF, while heavier injury might induce widespread pain via a higher and more chronic IL-6 release. The occurrence of widespread pain might be gated by the concentration and time course of CSF IL-6, which should be determined in further study. Moreover, recent research has intriguingly demonstrated that transferring CSF from young mice to old mice can restore memory function [40]. These findings expand our understanding of the potential therapeutic roles of CSF in neurological conditions. The long-reaching impact of CSF alterations, as indicated by our study, underscores the importance of exploring novel strategies for mitigating the systemic effects of tissue trauma, with implications not only for pain management but also for broader neurological health.

In our study, we applied a cytokine-focused antibody array for CSF analysis to evaluate the alteration of CSF in CCI-ION model. Combining the following bioinformatic analysis, the IL-6-STAT3 signaling was filtered. IL-6, a robust cytokine with wide-ranging biological functions, exerts its effects via interaction with the membrane-bound IL-6 receptor (mIL-6R) and the transducing membrane protein gp130. Classical IL-6 signaling pathways include JAK-STAT, mitogen-activated protein kinase/extracellular signal-regulated kinase (MAPK/ERK), and phosphatidylinositol 3-kinase/protein kinase B (PI3K/Akt) [41, 42]. IL-6 is predominantly induced as challenged by PGE2 and TNF-α in peripheral nerve injury, and is implicated in neuropathic pain via MAPK and STAT3 signaling [43–47]. Clinical studies have reported elevated IL-6 levels in the CSF in various pain-related neuropathological situations, including complex regional pain syndrome and lumbar radiculopathy [48–50]. Consistently, blocking IL-6/IL-6R has been proposed as a promising therapeutic strategy and leads to the development of several drugs [39, 51, 52]. Nonetheless, the role of IL-6 in neuropathological contexts exhibits considerable variability. For instance, in the serum-transferred K/BxN arthritis model, there was an observed increase in spinal IL-1β mRNA levels, whereas IL-6 levels remained unchanged, even though a trend towards higher IL-6 CSF levels was observed in rheumatoid arthritis patients [53]. Akin to our CSF transplant model, this murine model involves the transfer of serum containing autoantibodies from transgenic K/BxN mice to naive mice, leading to the induction of arthritis [54]. This suggests that although certain inflammatory pathways might be shared across diverse conditions, the principal inflammatory mediators within these pathways can significantly vary. This insight is particularly relevant when considering therapeutic interventions tailored to specific neuropathological conditions.

In this study, we further revealed the unique mode of widespread pain mediated by IL-6 in the CSF through astrocytic STAT3 activation in distant segments. We observed an upregulation of IL-6 at MDH and TG in CCI-ION model, suggesting that IL-6 could be secreted into the CSF by various cell types, including primary sensory neurons, microglia and astrocytes, and the exact cellular sources warrant further investigation. Moreover, nerve injury causes damage to the blood-spinal cord barrier, potentially allowing serum IL-6 and peripheral immune cells to contribute to CSF IL-6 levels [55]. Additionally, in our study, we specifically focused on identifying the expression of p-STAT3 in distal regions of SDH. While this analysis was limited to distal spinal areas, it is noteworthy that a previous study has shown activation of STAT3 at the injury site and sensory ganglia, which can subsequently drive IL-6 transcription [56]. This suggests a complex interplay where p-STAT3 may not only contribute to glia activation in distal spinal segments, but also potentially promote the IL-6 production in TG and MDH. Therefore, IL-6 released into the CSF can further activate STAT3, amplifying the inflammation responses and facilitating of the propagation of pain following peripheral nerve injury.

Apart from IL-6, elevated CSF leptin levels in CCI-ION rats emphasize its potential significance in widespread pain. In a clinical research, leptin has been reported to be associated with chronic widespread pain [57]. Moreover, patients with rheumatoid arthritis show considerably higher plasma levels of leptin contributing to low-grade inflammation [57–59]. Notably, leptin also exerts biological function via STAT3. Therefore, the contribution of CSF leptin to astrogliosis at distant segments and associated widespread pain should be addressed in further study. Our study also revealed a significant decrease in IL-13 levels in the CSF. IL-13, serving as an immune regulatory cytokine mainly secreted by activated Th2 cells, plays an important protective role in several inflammatory diseases and neuropathic pain by inhibiting the production of pro-inflammatory cytokines [60, 61]. In a clinical trial, a negative correlation has been observed between plasma IL-13 levels and the severity of pain in amputees with residual limb pain [62]. Therefore, it is reasonable to presume that reduced IL-13 in the CSF can further trigger the inflammatory response and contribute to widespread pain. Exogenous administration of IL-13 may hold therapeutic potential [63–65]. Additionally, the content of monocyte chemoattractant protein-1 (MCP-1) in CCI-ION’s CSF exhibited a trend towards elevation, though the changes did not reach statistical significance. MCP-1, as a key chemokine, regulates the migration and infiltration of monocytes/macrophages. Tissue and nerve damage can increase the expression of MCP-1 in both the injured sites and their innervated peripheral nervous system (PNS) and the content of MCP-1 in the CSF and serum is positively correlated, suggesting that MCP1 could be relevant for neuroimmune communication across the blood–brain barrier (BBB) [66–68]. Collectively, the peripheral inflammation may invade and contribute to the breakdown of BBB [69, 70]. This breakdown would further facilitate the infiltration of immune cells into the CNS along with inflammatory mediators, leading to their diffusion in the CSF [71, 72]. This process exaggerates the phenomenon of widespread pain. The only fly in the ointment is that detection at a single timepoint might also miss other critical information as the components in the CSF are dynamically changing. Higher throughput methods and longitudinal comparison at multiple timepoints can be applied in the future to solve this issue.

Glial cells activation is a pivotal mechanism underlying chronic pain [73]. In our study, CCI-ION induced the activation of microglia and astrocytes in distal segments of SDH. Interestingly, the widespread pain in the hind paws could only be alleviated by using astroglial toxins, while the inhibitor of microglia failed to produce a similar effect. Accumulating studies have underscored the critical role of microglia in the initiation of neuropathic pain, with its impact on established pain states being comparatively limited [74–76]. In contrast, astrocytes have been proved to exert a promoting effect on both the induction and maintenance of neuropathic pain [77–80]. Given that our intervention was conducted from POD 15 to POD 19 with effects observed on POD 21, it is understandable that astrocytes predominantly mediate chronic widespread pain, while acknowledging the potential contribution of microglia. Numerous studies have demonstrated the interaction between microglia and astrocytes. These activated glial cells can reciprocally activate each other by releasing various chemokines and inflammatory mediators, thereby synergistically contributing to chronic pain [81, 82].

In summary, the present study offers insights into the underlying mechanism of widespread pain induced by peripheral nerve injury, specifically focusing on the role of CSF containing IL-6. We observed that chronic constriction injury of infraorbital nerve induced the elevation of IL-6 in the CSF, subsequently activating astrocytic STAT3 signaling in distant segments of SDH and ultimately contributing to widespread pain. Interventions targeting the IL-6/STAT3 pathway demonstrated analgesic effects on widespread pain. Further investigation into this pathway may offer new strategies for improving the management and treatment of patients experiencing widespread pain.

## Conclusions

Our study demonstrates that cerebrospinal fluid IL-6 diffused original neuropathic pain to widespread pain in distant spinal segments via activating astrocyte STAT3 signaling.

### Supplementary Information

**Additional file 1: Fig. S1.** I.c. injection of IL-6 induced orofacial pain and widespread pain. **A** The workflow for i.c. injection of IL-6 for naïve rats. **B-D** The mechanical escape withdrawal threshold of vibrissal pad (**B**), the paw thermal withdrawal latency (**C**), and the paw mechanical withdrawal threshold (**D**) in rats receiving IL-6 (1.0 μg), IL-6 (5.0 μg) or vehicle. Two-way ANOVA, *p<0.05, **p<0.01, ***p<0.001, ****p<0.0001, n = 7/group. **Fig. S2.** Neutralizing IL-6 inhibited the acitvation and proliferation of astrocytes. Immunofluorescence staining of C3 (**A-B**), Ki67 (**C-D**), and p-STAT3 (**E-F**) in primary cultured astrocytes after incubation of CCI-ION's CSF with IL-6 antibody or isotype IgG, along with quantitative analysis. t test, ***p<0.001, ****p<0.0001, n = 9/group. Scale bar: 75 µm. 

**Additional file 2: Table S1**. List of antibodies for immunofluorescence staining.

**Additional file 3: Table S2.** List of statistic values for t test and ANOVA test.

## Data Availability

There are no data, software, databases, and application/tools available apart from those reported in the present study. All data are provided in the manuscript and supplementary data section.

## Abbreviations

AD: Alzheimer’s disease
BBB: Blood–brain barrier
BSA: Bovine serum albumin
CCI-ION: Chronic constriction injury of the infraorbital nerve
CDH: Cervical spinal dorsal horn
CNS: Central nervous system
CSF: Cerebrospinal fluid
C3: Complement 3
DMEM: Dulbecco’s modified eagle medium
ERK: Extracellular signal-regulated kinase
FBS: Fetal bovine serum
HMGB1: High mobility group box 1 protein
IL-6: Interleukin-6
IL-13: Interleukin-13
JAK-STAT: Janus kinase-signal transducers and activators of transcription
LAA: L-2-Aminoadipic acid
LDH: Lumbar spinal dorsal horn
MAPK: Mitogen-activated protein kinase
MDH: Medullary dorsal horn
mIL-6R: Membrane-bound IL-6 receptor
Mino: Minocycline
MCP-1: Monocyte chemoattractant protein-1
PBS: Phosphate buffered saline
p-IONX: Partial infraorbital nerve transection
PI3K/Akt: Phosphatidylinositol 3-kinase/protein kinase B
PNS: Peripheral nervous system
POD: Postoperative day
qRT-PCR: Quantitative real-time-polymerase chain reaction
SDH: Spinal dorsal horn
STAT3: Signal transducer and activator of transcription 3
TDH: Thoracic spinal dorsal horn
TG: Trigeminal ganglia
TLR4: Toll like receptor 4
