# Supplementary material for: IL-6 from cerebrospinal fluid causes widespread pain via STAT3-mediated astrocytosis in chronic constriction injury of the infraorbital nerve
Source: J Neuroinflammation. 2024 Feb 28;21:60. doi: 10.1186/s12974-024-03049-z (PMC10900663; doi:10.1186/s12974-024-03049-z)
Supplement: Supplementary file 1 — Additional file 1: Fig. S1. I.c. injection of IL-6 induced orofacial pain and widespread pain. A The workflow for i.c. injection of IL-6 for naïve rats. B-D The mechanical escape withdrawal threshold of vibrissal pad (B), the paw thermal withdrawal latency (C), and the paw mechanical withdrawal threshold (D) in rats receiving IL-6 (1.0 μg), IL-6 (5.0 μg) or vehicle. Two-way ANOVA, *p<0.05, **p<0.01, ***p<0.001, ****p<0.0001, n = 7/group. Fig. S2. Neutralizing IL-6 inhibited the acitvation and proliferation of astrocytes. Immunofluorescence staining of C3 (A-B), Ki67 (C-D), and p-STAT3 (E-F) in primary cultured astrocytes after incubation of CCI-ION's CSF with IL-6 antibody or isotype IgG, along with quantitative analysis. t test, ***p<0.001, ****p<0.0001, n = 9/group. Scale bar: 75 µm. [file 12974_2024_3049_MOESM1_ESM.docx]

**Additional file 1**

**
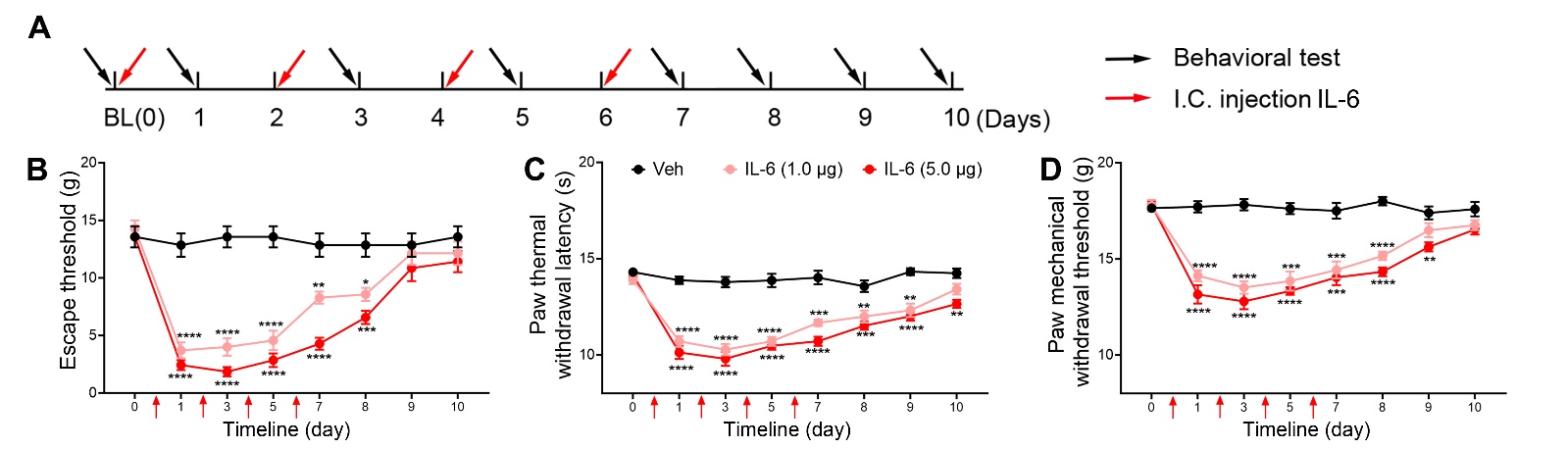
**

**Fig. S1.** I.c. injection of IL-6 induced orofacial pain and widespread pain. **A** The workflow for i.c. injection of IL-6 for naïve rats. **B-D** The mechanical escape withdrawal threshold of vibrissal pad (**B**), the paw thermal withdrawal latency (**C**), and the paw mechanical withdrawal threshold (**D**) in rats receiving IL-6 (1.0 μg), IL-6 (5.0 μg) or vehicle. Two-way ANOVA, **p<0.05*, ***p<0.01*, ****p<0.001*, *****p<0.0001*, n = 7/group.


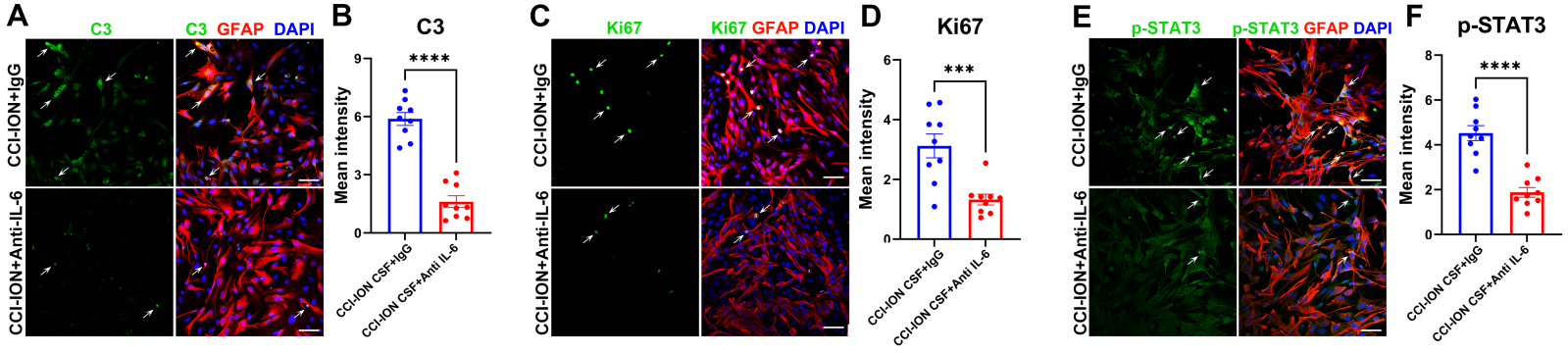


**Fig. S2.** Neutralizing IL-6 inhibited the activation and proliferation of astrocytes. **A-B** Immunofluorescence staining of C3 (**A-B**), Ki67 (**C-D**), and p-STAT3 (**E-F**) in primary cultured astrocytes after incubation of CCI-ION’s CSF with IL-6 antibody or isotype IgG, along with quantitative analysis. t test, ****p<0.001*, *****p<0.0001*, n = 9/group. Scale bar: 75 μm.
